# FAMILY INVOLVEMENT IN BRITISH COLUMBIA LTC FACILITIES DURING A PANDEMIC: THE IMPACT OF COVID-19 RESTRICTIONS

**DOI:** 10.1093/geroni/igad104.2836

**Published:** 2023-12-21

**Authors:** Tracy Christianson, Evans Appiah-Kusi, Jordan Bremner, Andrew Filewich, Amna Qazi, Colin Reid

**Affiliations:** Thompson Rivers University, Kamloops, British Columbia, Canada; Thompson Rivers University, Kamloops, British Columbia, Canada; Thompson Rivers University, Kamloops, British Columbia, Canada; University of British Columbia - Okanagan, Kelowna, British Columbia, Canada; Thompson Rivers University, Kamloops, British Columbia, Canada; University of British Columbia - Okanagan, Kelowna, British Columbia, Canada

## Abstract

Family involvement in long-term care is linked directly to LTC resident quality of life and family satisfaction with care. The public health restrictions implemented to protect the vulnerable LTC population, the majority of whom have some type and level of dementia, represented a serious breach of families’ ability to visit and care for their institutionalized loved ones. While much of the early research on the impact of the restrictions focused on the LTC resident and the paid care staff, the purpose of this study was to explore what impacts COVID-19 restrictions had on family involvement and family well-being in LTC facilities across the province of British Columbia. A mixed-methods design using a survey, interviews, and arts-based focus groups was the approach implemented. Families (direct, friends, legal guardians) of residents living in long-term care settings were invited to participate. Using a nested approach, online and paper-based surveys (N=55) were distributed, one-to-one interviews conducted (N=19), ending with two arts-based focus groups (n=4 each focus group). Qualitative interview data revealed six different but interconnected themes through the thematic analysis of the data. The themes were: 1) Quality of life, 2) Quality of care, 3) Mental Health concerns, 4) Communication, 5) Communication Strategies, and 6) The Rules. Quantitative results indicated positive relationships between satisfaction with care and a series of variables that aligned with the qualitative themes (example: satisfaction and treatment of family by LTC staff). Strategies offered by family participants to improve public health policies for future outbreaks are reviewed.

